# Predictors of Mortality in Adults with Acute Kidney Injury Requiring Dialysis: A Cohort Analysis

**DOI:** 10.1155/2022/7418955

**Published:** 2022-09-12

**Authors:** Charles Kangitsi Kahindo, Olivier Mukuku, Vieux Momeme Mokoli, Ernest Kiswaya Sumaili, Stanis Okitotsho Wembonyama, Zacharie Kibendelwa Tsongo

**Affiliations:** ^1^Department of Internal Medicine, Faculty of Medicine, University of Goma, Goma, Democratic Republic of the Congo; ^2^Clinique Internationale de Médecine Avancé Au Kivu, Goma, Democratic Republic of the Congo; ^3^Institut Supérieur des Techniques Médicales de Lubumbashi, Lubumbashi, Democratic Republic of the Congo; ^4^Department of Nephrology, Faculty of Medicine, University of Kinshasa, Kinshasa, Democratic Republic of the Congo; ^5^Department of Pediatrics, Faculty of Medicine, University of Lubumbashi, Lubumbashi, Democratic Republic of the Congo; ^6^Department of Internal Medicine, Faculty of Medicine, University of Kisangani, Kisangani, Democratic Republic of the Congo

## Abstract

**Introduction:**

Acute kidney injury (AKI) requiring renal replacement therapy is accompanied by considerable mortality. This present study evaluated predictors of mortality at initiation of hemodialysis (HD) in AKI patients in Goma (in the Democratic Republic of the Congo (DRC)).

**Methods:**

A single-centre cohort survey evaluated the clinical profile and survival rates of AKI patients admitted to HD in the only HD centre in Goma, North Kivu province (DRC). Data were collected from patients who underwent HD for AKI. Patient demographics, comorbidities, clinical presentation, laboratory tests, and mortality were reviewed and analyzed. The survival study used the Kaplan–Meier curve. Predictors of mortality were evaluated using Cox regression.

**Results:**

Of the 131 eligible patients, the mean age was 43.69 ± 16.56 years (range: 18–90 years). Men represented 54.96% of the cohort. The overall HD mortality rate was 25.19% (*n* = 33). In multivariate analysis, independent predictors of mortality in AKI stage 3 patients admitted to HD were as follows: age ≥ 60 years (adjusted hazard ratio (AHR) = 15.89; 95% CI: 3.98–63.40; *p* < 0.0001), traditional herbal medicine intake (AHR = 5.10; 95% CI: 2.10–12.38; *p* < 0.0001), HIV infection (AHR = 5.55; 95% CI: 1.48–20.73; *p*=0.011), anemia (AHR = 9.57; 95% CI: 2.08–43.87; *p*=0.004), hyperkalemia (AHR = 6.23; 95% CI: 1.26–30.72; *p*=0.025), respiratory distress (AHR = 4.66; 95% CI: 2.07–10.50; *p* < 0.0001), and coma (AHR = 11.39; 95% CI: 3.51–36.89; *p* < 0.0001).

**Conclusion:**

Initiation of hemodialysis with AKI has improved survival in patients with different complications.

## 1. Introduction

Acute kidney injury (AKI) is defined as a sudden and often reversible decline in kidney function. It is a common complication in hospitalized patients and is strongly associated with increased morbidity, mortality, and healthcare costs [1, 2]. AKI is responsible for a high number of deaths in adults worldwide, especially as recent studies do not show a trend towards a decrease in the incidence and mortality [2, 3]. The disease is underestimated due to difficulties in accessing care, lack of knowledge of the general demographics, and lack of blood tests in hospitals [4].

As no specific pharmacological protocol is effective in patients with AKI, their management is limited to replacement therapy in which renal replacement therapy (RRT) plays a central role [5]. RRT, as a replacement therapy, remains the main treatment strategy for patients with AKI in KDIGO stage 3. Patients on maintenance hemodialysis (MHD) are at an increased risk of several complications such as associated infections and high mortality from the beginning of treatment initiation [4]. MHD performs some essential renal functions by correcting water and electrolyte balance, achieving superior acid-base homeostasis, and more efficiently removing uremic toxins [6].

It has been suggested that AKI cases in developing countries such as the Democratic Republic of the Congo (DRC) may have a different epidemiological profile to those in developed countries. AKI patients in developing countries tend to be younger, have AKI without many risk factors, have fewer comorbidities, and have a lower mortality rate [2, 7–9].

Thus, studying the profile of AKI patients receiving hemodialysis (HD) will expand the collection of AKI-related data in low-income countries and contribute to decision-making efforts in the region. The objective of this study was to assess mortality and its associated factors in patients with AKI stage 3 undergoing HD in Goma city in the DRC.

## 2. Materials and Methods

### 2.1. Study Design and Population

This is a cohort study from January 2019 to December 2021 conducted in the only hemodialysis centre of CIMAK (Clinique Internationale de Médecine Avancée au Kivu) in Goma in the province of North Kivu (DRC), which has 4 Fresenius 5008 S CorDiax dialysis machines (Fresenius Medical Care, Bad Homburg vor der Höhe, Germany). This centre receives patients from all hospitals in the province and even from neighbouring provinces such as Ituri, Haut-Uélé, South-Kivu, Maniema, and Tshopo.

We used the KDIGO stages for the indication of hemodialysis, of which stage 3 (an increase in serum creatinine greater than or equal to 4.0 mg/dL (353.60 micromoles/L) or a 3-fold increase in the initial value [9]), sequentially including all patients with a diagnosis of AKI admitted to our dialysis centre. Patients were enrolled if they were 18 years old or over, had a creatinine level under 4 mg/dL, and had anuria or oliguric disorders without signs of chronicity. They were then monitored for a total of 30 days until they were discharged or dead. Patients unable to give verbal or written informed consent were not included. This study did not include patients associated with chronic renal disease.

### 2.2. Determining the Sample Size

The proportion of patients with AKI stage 3 was 12.8%, as reported in a previous study on adult AKI patients in the DRC [9]. The required sample size was determined using the formula: *n* = *z*^2^*pq*/*d*^2^ or *n* = desirable sample size, *z*^2^ = 1.96 (critical value at the 95% significance level),  *p*=0.128 (proportion of patients with AKI stage 3), *q* = (1 − *p*), and *d* = 0.05 (acceptable marginal error).

The calculated sample size was 172 patients. Based on the inclusion criteria, 41 patients were excluded (19 did not consent, 14 had underlying chronic kidney disease, and 8 were not AKI stage 3). Thus, 131 patients were finally included in the study.

### 2.3. Data Collection

A structured questionnaire was developed from the previous AKI studies [9–11]. We collected information on patient demographics (age, gender, and occupation), diagnosis, etiological factors of AKI (hypertension, diabetes mellitus, smoking, alcohol consumption, use of indigenous products, surgery, and infection), laboratory examinations (repeated measurements of creatinine, urea, electrolytes, complete blood count, HIV serology, etc.), and mode of discharge.

AKI has been defined according to the KDIGO 2012 classification criteria by stages of AKI [12]. According to the WHO, anemia was defined as a hemoglobin level of less than 13 g/dL in men and less than 12 g/dL in women [13]. Hyperkalemia was defined as plasma potassium > 5.5 mmol/L. It is moderate between 6.1 and 6.9 mmol/L and severe if potassium is >7 mmol/L [14]. The main outcome variable was mortality in 30 days of follow-up (short-term), defined as death before hospital discharge. The parameters used for prediction were all measured before dialysis.

### 2.4. Statistical Analysis

The data collected were entered and coded in Microsoft Excel, and statistical analyses were performed using STATA version 16.0. Descriptive analysis was performed using calculations of proportions for qualitative variables (frequencies and percentages) and means and standard deviations (SD) for quantitative variables. Student's t-test was used to compare the means of laboratory tests between deceased patients and survivors. Cross-tabulations were used to compare proportions of categorical variables. The Cox regression model was fitted to determine independent predictors of mortality. The Kaplan–Meier curves were used to analyze the 30-day overall survival of cases. The survival comparison of different subgroups used the log-rank test. All cases with no event of interest (death) at the end of the follow-up period were censored. We considered a probability of error of less than 0.05 as a significance level.

### 2.5. Ethical Considerations

The Medical Ethics Committee of the University of Goma approved the study (approval number: UNIGOM/CEM/002/2021), and permission for data collection was obtained from the administration of the CIMAK hemodialysis centre. Written or oral informed consent was obtained from all participants, and the data collected were confidential and anonymous.

## 3. Results

A total of 131 patients with AKI stage 3 were analyzed. The demographic and clinical characteristics of the patients are summarized in Table 1. The mean age was 43.69 ± 16.56 years (range: 18–90 years). Patients aged 30–39 years were 24.43%. Men accounted for 72 (54.96%). Almost 30% of the patients drank alcohol, and 12.21% smoked tobacco. The clinical picture was dominated by coma (62.59%), anuria (49.61%), vomiting (38.17%), pulmonary edema (33.59%), respiratory distress (25.19%), and seizures (19.08%); 41.33% of the patients had hyperkalemia. The overall in-hospital mortality rate in HD was 25.19% (*n* = 33). In bivariate analysis, age ≥ 60 years, being unemployed, anuria, coma, respiratory distress, and hyperkalemia were significantly associated with mortality in AKI stage 3 patients admitted to HD.

Table 2 shows that the etiological factors of AKI found were anemia (67.18%), nephrotoxic drugs (59.54%), hypertension (57.25%), traditional herbal medicine intake (45.04%), malaria (43.51%), sepsis (32.06%), surgery (24.43%), diabetes mellitus (22.90%), gastroenteritis (19.85%), COVID-19 infection (7.63%), HIV infection (5.34%), and sickle cell disease (3.05%). In bivariate analysis, anemia, indigenous products, and HIV infection were significantly associated with mortality in AKI stage 3 patients admitted to HD.

Table 3 shows the comparison of laboratory values between deceased and survivors. We find that creatinine (10.33 ± 3.79 vs. 8.08 ± 3.76; *p*=0.0036), urea (318.68 ± 91.43 vs. 257.77 ± 92.69; *p*=0.0014), potassium (6.85 ± 1.07 vs. 5.18 ± 1.28; *p* < 0.0001), and white blood cells (13168.79 ± 7371.62 vs 8491.33 ± 7495.71; *p* < 0.0001) were significantly higher in deceased than in survivors. In contrast, the mean values for hemoglobin (8.09 ± 1.72 vs. 10.85 ± 2.10; *p* < 0.0001), sodium (127.60 ± 5.98 vs. 133.64 ± 7.51; *p* < 0.0001), and bicarbonate (14.82 ± 6.08 vs. 23.81 ± 15.61; *p*=0.0016) were significantly lower in deceased than those in survivors. No statistical differences were found when comparing the mean values of blood glucose (141.95 ± 90.76 vs 138.11 ± 71.11; *p*=0,8033) and C-reactive protein (32.42 ± 22.38 vs 26.07 ± 24.24; *p*=0.2147).

**Figure 1:**
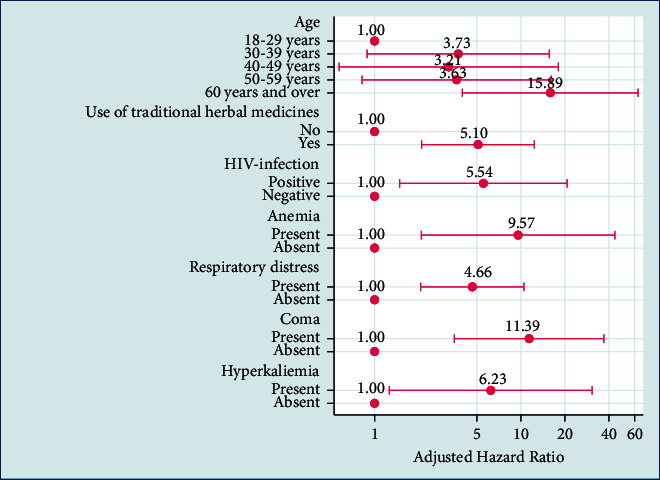
Cox multivariate regression analysis of independent predictors of mortality in AKI stage 3 patients up to 30 days admitted to the hemodialysis unit.

After Cox regression, the independent predictors of mortality in AKI stage 3 patients admitted to hemodialysis were age ≥ 60 years (adjusted hazard ratio (AHR) = 15.89; 95% CI: 3.98–63.40; *p* < 0.0001), traditional herbal medicine intake (AHR = 5.10; 95% CI: 2.10–12.38; *p* < 0.0001), HIV-infection status (AHR = 5.55; 95% CI: 1.48–20.73; *p*=0.011), anemia (AHR = 9.57; 95% CI: 2.08–43.87; *p*=0.004), hyperkalemia (AHR = 6.23; 95% CI: 1.26–30.72; *p*=0.025), respiratory distress (AHR = 4.66; 95% CI: 2.07–10.50; *p* < 0.0001), and coma (AHR = 11.39; 95% CI: 3.51–36.89; *p* < 0.0001) (Figure 1).

**Figure 2:**
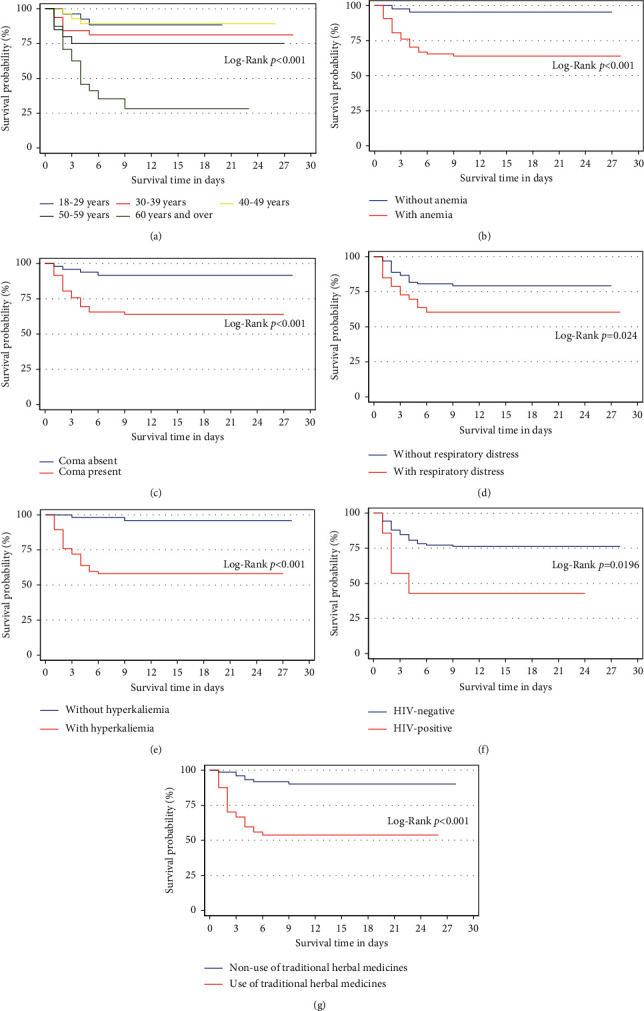
Kaplan–Meier analysis showing the probability of survival in AKI stage 3 patients up to 30 days according to: (a) age; (b) presence of anemia; (c) presence of coma; (d) presence of respiratory distress; (e) presence of hyperkalemia; (f) presence of HIV infection; (g) use of traditional herbal medicines.

As shown by Kaplan–Meier curves (Figure 2), the risk of death in patients included in this study increased with age ≥ 60 years (*p* < 0.001), traditional herbal medicine intake (*p* < 0.001), HIV infection (*p*=0.0196), anemia (*p* < 0.001), hyperkalemia (*p* < 0.001), respiratory distress (*p*=0.024), and coma (*p* < 0.001).

## 4. Discussion

This single-centre cohort study presented the general characteristics of a sample of patients with KDIGO stage 3 AKI in the HD unit and assessed the impact on mortality at the time of initiation of ERT. Given that AKI is a major health problem and exhibits a silent progression in hospitalized patients, the risk of mortality increases exponentially in accordance with associated risk factors. The present study of AKI patients requiring dialysis has shed light on the causes and prognosis of these patients in the Congolese setting.

The mortality rate in HD patients with AKI varies between hospitals and between countries. This study reported that among 131 patients admitted to HD, 33 (25.19%) had died. Similar figures have been found in studies of other countries such as Burundi (17.4%) [15], Côte d'Ivoire (26.2%) [16], Burkina Faso (24%) [17], Nigeria (28.8%) [18], Ethiopia (29.1%) [19], and South Africa (31%) [20]. On the other hand, higher rates than ours were reported in Egypt (30% to 60%) [21], the United States (39%) [22], Japan (62%) [23], and Sudan (91%) [24]. This difference can be explained by a number of factors, including the sociodemographic characteristics of the study population. For example, the patients in our study, like those in the study by Lengani et al. [17], were young compared to those in developed countries whose age was generally over 60 years [23]. This difference in the mortality rate could also be due to geographical factors, comorbidities, the method of selection of participants, and the time of initiation of dialysis.

The present study investigated the determinants of HD initiation mortality in patients with AKI, incorporating as many risk factors as possible. The results of multivariate logistic regression analysis identified age ≥ 60 years, traditional herbal medicine intake, HIV infection, anemia, hyperkalemia, respiratory distress, and coma as predictors of mortality in AKI patients requiring HD. Several studies reported the association between advanced age and mortality in patients with AKI in HD [10, 25]. As in the present study, a US study of 725 AKI patients who received HD reported that age over 60 years was an independent risk factor for in-hospital mortality [26]. In an Irish study, age 75 and over was a predictor of mortality [27]. This could be explained by the presence of several comorbidities in the elderly. On the other hand, other studies did not observe significant differences between older and younger patients with regard to mortality in HD [28, 29].

The use of traditional herbal medicines in our cohort also appears to be a risk factor for death in HD patients with AKI. Some studies have also shown this direct correlation between the AKI occurrence and the traditional herbal medicine intake. In South Africa, Luyckx et al. [30] found that although a proportion of patients with underlying systemic or renal conditions may contribute to renal dysfunction, in the majority of cases, the use of traditional herbal medicines appeared to be the immediate and most likely cause. These authors found an overall mortality in AKI patients of 41%. We know that the use of traditional herbal medicines is common in Africa, and most patients consulting traditional healers do not need to use western medicine as a first line of treatment; they only go to hospital when the clinical signs worsen. AKI is one of the most serious complications resulting from the use of traditional plants, accounting for about 35% of all AKI cases in Africa. Little is known about the nephrotoxicity of plants used in traditional medicine in Africa because their chemical composition is not known in most cases [31]. In sub-Saharan Africa, traditional practitioners have their own basis for diagnosis, which is mostly symptomatic but claims to treat a variety of diseases. Traditional medicine is totally outside the rational approach, empiricism being predominant [32].

HIV infection appears in our study as a risk factor for mortality at HD initiation, which Camara and Chothia [20] confirmed in their study that HIV was found to be a mortality factor among people living with HIV with AKI admitted to HD. It should be remembered that HIV infection leads to inflammation, which can disrupt the function of the kidneys (and other organ systems). In addition, patients with AKI related to HIV infection may appear weak, dehydrated, and are exposed to certain drugs (antibiotics, antifungal agents, etc.) that can impair kidney function. It is likely that all these factors contribute to the poor general condition of patients admitted to HD units [33].

The present study showed that respiratory distress is a risk factor for death in HD patients. This finding is consistent with findings of some studies showing that respiratory distress associated with AKI increases mortality. McNicholas et al. [34] found that hospital mortality increased by 31% in patients with acute respiratory distress syndrome without AKI, by 50% with mild/moderate AKI (*p* ≤ 0.001 compared to no acute kidney injury) and 58% with severe AKI (*p* ≤ 0.001 compared to no acute kidney injury and mild/moderate acute kidney injury). Several pathophysiological mechanisms are involved, including renal hypoperfusion related to mechanical ventilation, hypoxemia, sepsis, systemic inflammation, and cytokine storm [35] as well as direct toxicity of the virus on proximal tubular cells and podocytes, mediated by angiotensin-converting receptor 2 (ACE 2) and transmembrane serine protease 2. Dialysis is made difficult at the hypercoagulable state in patients with SARS-CoV-2, which causes early filter thrombosis leading to death [36]. In cases where AKI and acute respiratory distress syndrome are combined, mortality and length of stay in intensive care units increase and management becomes complex.

Organ failure is often a cause of death in AKI. The present study found that coma, which is also a sign of organ failure, is a risk factor for death in AKI patients requiring HD. Many studies reported that AKI was associated with widespread organ dysfunction such as the lungs, heart, brain, and liver. The study by Paškevičius et al. showed that organ dysfunction was statistically significantly associated with death [37]. The rationale for this is that the physiological and molecular mechanisms of distant organ interactions in AKI, including leukocyte activation and infiltration, production of soluble factors such as inflammatory cytokines/chemokines, and endothelial injury, are well documented. Oxidative stress and reactive oxygen species (ROS) production as well as deregulation of cell death in distant organs are also important mechanisms of AKI-induced distant organ dysfunction [38].

We found anemia as a risk factor for death in patients admitted to HD in the present study. Some studies did not find anemia as a factor for death; for example, Guei et al. [15] did not find anemia as a risk factor for death, but patients with Hb ˂ 8 g/dL appeared to have a better vital prognosis. This difference would probably be explained by patient selection and time of initiation of HD.

The occurrence of AKI can be life threatening due to hyperkalemia, which is a risk factor for death at the start of HD. As in the present study, this finding has been noted in previous studies [11]. The reason for this is that acute hyperkalemia leads to conduction disturbances in muscle cells and neurons. These disturbances lead to an increase in repolarization time and a decrease in intraventricular conduction in myocytes, resulting in rhythm disturbances that may ultimately lead to death. Therefore, it is important to emphasize the prevention of AKI and make AKI available to the population to avoid high mortality.

As a limitation of the study, first, we emphasize that the study suffers from its retrospective nature, being limited to determining the relationship between the risk factor studied and mortality. Second, the paraclinical explorations are at the expense of the patients and are not very exhaustive, the nephrological history of our patients is not well known, and the therapeutic manipulations in the health facilities of origin contributed to a complex clinical picture, which integrates AKI, its causes and consequences. The fact that our study is monocentric does not allow us to generalize our results to the whole country.

Nevertheless, the present study has the merit of being one of the first on AKI carried out in the eastern DRC to show the mortality factors at the initiation of dialysis of patients with AKI.

## 5. Conclusion

Our study showed that AKI has a high mortality rate (25.19%), although it improves with the initiation of RRT if started earlier. It also identified risk factors for death in AKI patients admitted to HD. More effort should be devoted to the prevention of AKI, including improved treatment of aetiologies, timely diagnosis and management of complications, and facilitating access to RRT before death occurs.

## Figures and Tables

**Table 1:** Demographic and clinical characteristics of AKI stage 3 Congolese adults admitted to the CIMAK hemodialysis centre in North-Kivu province in the DRC.

Variable	Total (*N* = 131)	Dead (*n* = 33)		Survivors (*n* = 98)		HR [CI95%]	*p* value
Age							
18–29 years	27	3	11.11%	24	88.89%	Reference	
30–39 years	32	6	18.75%	26	81.25%	1.77 [0.44–7.07]	0.420
40–49 years	28	3	10.71%	25	89.29%	0.95 [0.19–4.71]	0.950
50–59 years	20	5	25.00%	15	75.00%	2.52 [0.60–10.54]	0.207
≥60 years	24	16	66.67%	8	33.33%	7.85 [2.28–27.03]	0.001
Gender							
Female	59	11	18.64%	48	81.36%	Reference	
Male	72	22	30.56%	50	69.44%	1.78 [0.86–3.67]	0.119
Occupation							
Private sector	46	6	13.04%	40	86.96%	Reference	
Public sector	26	7	26.92%	19	73.08%	2.20 [0.74–6.54]	0.157
Unemployed	59	20	33.90%	39	66.10%	2.75 [1.10–6.86]	0.030
Alcohol consumption							
No	93	24	25.81%	69	74.19%	Reference	
Yes	38	9	23.68%	29	76.32%	0.92 [0.43–2.00]	0.839
Smoking							
No	115	30	26.09%	85	73.91%	Reference	
Yes	16	3	18.75%	13	81.25%	0.68 [0.21–2.24]	0.532
Anuria							
No	66	11	16.67%	55	83.33%	Reference	
Yes	65	22	33.85%	43	66.15%	2.11 [1.02–4.36]	0.043
Coma							
No	49	4	8.16%	45	91.84%	Reference	
Yes	82	29	35.37%	53	64.63%	5.01 [1.76–14.27]	0.003
Respiratory distress							
No	98	20	20.41%	78	79.59%	Reference	
Yes	33	13	39.39%	20	60.61%	2.15 [1.07–4.32]	0.032
Seizures							
No	106	27	25.47%	79	74.53%	Reference	
Yes	25	6	24.00%	19	76.00%	0.97 [0.40–2.36]	0.956
Pulmonary edema							
No	87	18	20.69%	69	79.31%	Reference	
Yes	44	15	34.09%	29	65.91%	1.73 [0.87–3.44]	0.116
Vomiting							
No	81	23	28.40%	58	71.60%	Reference	
Yes	50	10	20.00%	40	80.00%	0.68 [0.32–1.43]	0.312
Hyperkalemia							
No	56	2	96.43%	54	96.43%	Reference	
Yes	75	31	41.33%	44	58.67%	14.38 [3.44–60.15]	0.0001

**Table 2:** Causes of acute kidney injury among AKI stage 3 Congolese adults admitted to the CIMAK hemodialysis centre in North-Kivu province in the DRC.

Variable	Total (*N* = 131)	Dead (*n* = 33)		Survivors (*n* = 98)		HR [CI95%]	*p* value
Anemia							
No	43	2	4.65%	41	95.35%	Reference	
Yes	88	31	35.23%	57	64.77%	8.94 [2.14–37.41]	0.003
Surgery							
No	99	27	27.27%	72	72.73%	Reference	
Yes	32	6	18.75%	26	81.25%	0.62 [0.26–1.50]	0.291
COVID-19							
No	121	28	23.14%	93	76.86%	Reference	
Yes	10	5	50.00%	5	50.00%	2.52 [0.97–6.53]	0.058
Diabetes mellitus							
No	101	25	24.75%	76	75.25%	Reference	
Yes	30	8	26.67%	22	73.33%	1.11 [0.50–2.47]	0.792
Sickle cell disease							
No	127	31	24.41%	96	75.59%	Reference	
Yes	4	2	50.00%	2	50.00%	2.32 [0.55–9.70]	0.250
Gastroenteritis							
No	105	24	22.86%	81	77.14%	Reference	
Yes	26	9	34.62%	17	65.38%	1.65 [0.76–3.55]	0.201
Hypertension							
No	56	17	30.36%	39	69.64%	Reference	
Yes	75	16	21.33%	59	78.67%	0.68 [0.34–1.35]	0.272
Traditional herbal medicine intake							
No	74	7	9.46%	67	90.54%	Reference	
Yes	57	26	45.61%	31	54.39%	6.12 [2.65–14.15]	0.000
Nephrotoxic drugs							
No	53	15	28.30%	38	71.70%	Reference	
Yes	78	18	23.08%	60	76.92%	0.78 [0.39–1.55]	0.483
Malaria							
No	72	20	27.78%	52	72.22%	Reference	
Yes	59	13	22.03%	46	77.97%	0.76 [0.38–1.52]	0.437
Sepsis							
No	89	22	24.72%	67	75.28%	Reference	
Yes	42	11	26.19%	31	73.81%	1.16 [0.56–2.39]	0.687
HIV infection							
No	124	29	23.39%	95	76.61%	Reference	
Yes	7	4	57.14%	3	42.86%	3.16 [1.11–9.04]	0.031

**Table 3:** Comparison of the mean values for laboratory investigations between deceased and survivors at the CIMAK hemodialysis centre in North-Kivu province in the DRC.

Variable	Total (*N* = 131)	Dead (*n* = 33)	Survivors (*n* = 98)	*p* value^*∗*^
Creatinine (mg/dL)	8.65 ± 3.88	10.33 ± 3.79	8.08 ± 3.76	0.0036
Urea (mg/dL)	273.11 ± 95.78	318.68 ± 91.43	257.77 ± 92.69	0.0014
Potassium (mEq/L)	5.60 ± 1.42	6.85 ± 1.07	5.18 ± 1.28	<0.0001
White blood cells (elements/mm^3^)	9669.62 ± 5994.30	13168.79 ± 7371.62	8491.33 ± 7495.71	<0.0001
Hemoglobin (g/dL)	10.15 ± 2.34	8.09 ± 1.72	10.85 ± 2.10	<0.0001
Sodium (mEq/L)	132.12 ± 7.60	127.60 ± 5.98	133.64 ± 7.51	<0.0001
Bicarbonate (mmol/L)	21.54 ± 14.36	14.82 ± 6.08	23.81 ± 15.61	0.0016
C-reactive protein (g/L)	27.67 ± 25.37	32.42 ± 22.38	26.07 ± 24.24	0.2147
Blood glucose (mg/dL)	139.08 ± 76.18	141.95 ± 90.76	138.11 ± 71.11	0.8033

^*∗*^Student's *t*-test.

## Data Availability

The datasets used to support the findings of this study are available from the corresponding author upon request.

## Abbreviations

95% CI: 95% confidence intervals
AKI: Acute kidney injury
AHR: Adjusted hazard ratio
CKD: Chronic kidney disease
COVID-19: Coronavirus 2019
DRC: Democratic Republic of the Congo
HD: Hemodialysis
HIV: Human immunodeficiency virus
KDIGO: Kidney Disease: Improving Global Outcomes
RRT: Renal replacement therapy
SSA: Sub-Saharan Africa.
